# Oxytocin Receptor Exon III Methylation in the Umbilical Cord Blood of Newborns With Prenatal Exposure to Crack Cocaine

**DOI:** 10.3389/fcell.2021.639287

**Published:** 2021-06-04

**Authors:** Talita Baptista, Lucas Araújo de Azeredo, Aline Zaparte, Thiago Wendt Viola, Sayra Catalina Coral, Maria Aparecida Nagai, Flávia Rotea Mangone, Ana Carolina Pavanelli, Jaqueline B. Schuch, Victor Mardini, Claudia M. Szobot, Rodrigo Grassi-Oliveira

**Affiliations:** ^1^Developmental Cognitive Neuroscience Lab, School of Medicine, Brain Institute of the Rio Grande do Sul (InsCer), Pontifícia Universidade Católica do Rio Grande do Sul (PUCRS), Porto Alegre, Brazil; ^2^Discipline of Oncology, Department of Radiology and Oncology, Faculty of Medicine, University of São Paulo, São Paulo, Brazil; ^3^Laboratory of Molecular Genetics, Center for Translational Research in Oncology, Cancer Institute of the State of São Paulo, São Paulo, Brazil; ^4^Graduate Program in Psychiatry and Behavioral Sciences, Universidade Federal do Rio Grande do Sul, Porto Alegre, Brazil; ^5^Child and Adolescent Psychiatry Service (SPIA), Hospital de Clínicas de Porto Alegre (HCPA), Porto Alegre, Brazil; ^6^Translational Neuropsychiatry Unit, Department of Clinical Medicine, Aarhus University, Aarhus, Denmark

**Keywords:** cocaine, substance use disorders, OXTR, epigenetic, prenatal, pregnancy, pyrosequencing, newborn

## Abstract

**Background:**

Prenatal cocaine exposure (PCE) is associated with behavioral, cognitive, and social consequences in children that might persist into later development. However, there are still few data concerning epigenetic mechanisms associated with the effects of gestational cocaine exposure, particularly in human newborns.

**Aims:**

We investigated the effects of PCE on DNA methylation patterns of the Oxytocin Receptor (*OXTR*) gene in the umbilical cord blood (UCB). The relationship between UCB DNA methylation levels and the severity of the mother’s cocaine use during pregnancy was also evaluated.

**Methods:**

In this cross-sectional study, 28 UCB samples of newborns with a history of crack cocaine exposure *in utero* and 30 UCB samples of non-exposed newborns (NEC) were compared for DNA methylation levels at two genomic loci located in exon III of the *OXTR* gene (OXTR1 and OXTR2) through pyrosequencing. Maternal psychopathology was investigated using the Mini International Neuropsychiatric Interview, and substance use characteristics and addiction severity were assessed using the Smoking and Substance Involvement Screening Test (ASSIST).

**Results:**

No differences between newborns with a history of PCE and NEC were observed in OXTR1 or OXTR2 DNA methylation levels. However, regression analyses showed that maternal addiction severity for crack cocaine use predicted OXTR1 DNA methylation in newborns.

**Conclusion:**

These data suggest that *OXTR* methylation levels in the UCB of children are affected by the severity of maternal crack cocaine usage. Larger studies are likely to detect specific changes in DNA methylation relevant to the consequences of PCE.

## Introduction

Crack cocaine, a smoked form of cocaine, is a highly addictive drug (Dias et al., 2011). Crack-cocaine consumption during pregnancy is an even greater public health concern, because both the mother and baby are vulnerable to the negative effects of the drug (Bastos and Bertoni, 2014). Furthermore, prenatal cocaine-exposed (PCE) children can develop impairments in memory function (Frazer et al., 2018), attention (Carmody et al., 2011; Beeghly et al., 2014), social function (Williams and Johns, 2014), and affiliative behaviors (Fragkaki et al., 2019).

Prenatal cocaine exposure stimulates the fetal central nervous system, acting mainly on the dopaminergic system (Gaccioli and Lager, 2016; Gkioka et al., 2016). Oxytocin (OT) and dopamine have overlapping pathways, and this shared neurobiology results in the bidirectional communication between dopaminergic and oxytocinergic systems, with implications for reward-related behaviors (Lee et al., 2016). OT is produced in the hypothalamus and has been associated with social and affiliative behaviors, such as pair-bonding (Feldman and Bakermans-Kranenburg, 2017), parent–infant bonding (Keebaugh et al., 2015), social memory (Lin and Hsu, 2018), empathy (Ebert and Brüne, 2018), and drug addiction (Baracz and Cornish, 2013). Evidence suggests that in addition to long-term neuroadaptations in brain OT system induced by chronic cocaine exposure, manipulations that increase OT levels might improve drug withdrawal symptoms (Williams and Johns, 2014). For instance, the release of OT during pair-bonding formation or parental behavior has been shown to decrease the rewarding properties of drug dependence (Liu et al., 2010, 2011). Particularly during gestation, cocaine exposure alters the production of OT in neonates, as well as its receptor number and binding affinity (Johns et al., 2004). Therefore, OT signaling and behaviors associated with OT effects can be derailed by cocaine abuse during pregnancy.

Oxytocin mediates its effects by binding with the OT receptor encoded by the *OXTR* gene (Chr:3p25.3), a polypeptide of 389 amino acids with seven transmembrane domains. The OXTR is a class I G coupled receptor. The *OXTR* gene spans 17 kb and contains three introns and four exons. Exons I and II correspond to the 5′-non-coding region, followed by exons III and IV, encoding the amino acids of the receptor (Gimpl and Fahrenholz, 2001). Exon III starts 142 bp upstream from the adenosine of the ATG initiation codon and spans 922 bp downstream encoding beyond the sixth transmembrane domain of the receptor (Inoue et al., 1994). Additionally, accumulating evidence suggests that epigenetic modifications at the exon III critically contribute to *OXTR* gene expression regulation (Kumsta et al., 2013; Dadds et al., 2014; Cappi et al., 2016).

Epigenetic variability in the *OXTR* gene provides regulation of the oxytocin system in response to environmental events, especially those occurring during early childhood (Unternaehrer et al., 2015). This is often related to an increased risk of developing mental disorders later in life (Mehta et al., 2016; Ajonijebu et al., 2017; Kraaijenvanger et al., 2019). One of the most commonly studied epigenetic modifications is DNA methylation. This epigenetic mechanism consists of chemical modifications by the covalent attachment of methyl groups to cytosines (Cecil et al., 2014; Mehta et al., 2016). In mammalian cells, DNA methylation primarily occurs at cytosines that precede a guanine nucleotide at loci known as cytosine-phosphate-guanine (CpG) sites (Breton-Larrivée et al., 2019). Genomic regions rich in CpGs are called “CpG islands” and one of them can be found in the *OXTR* gene within exons I–III (Gimpl and Fahrenholz, 2001; Kumsta et al., 2013; Mehta et al., 2016). Using human blood samples, studies have associated *OXTR* CpG island methylation with child conduct disorder (Dadds et al., 2014), obsessive-compulsive disorder (Cappi et al., 2016), social behaviors (Jack et al., 2012), anxiety disorders, depression (Chagnon et al., 2015), and autism spectrum disorder (Gregory et al., 2009). However, no previous study has demonstrated the relationship between prenatal cocaine exposure and *OXTR* gene methylation.

Given the relationship between OT, addiction, and social behavior, the present study aimed to evaluate *OXTR* exon III DNA methylation levels in the umbilical cord blood (UCB) of prenatal crack cocaine-exposed newborns and compare these to those in non-exposed newborns. The relationship between UCB DNA methylation levels and the severity of the mother’s cocaine use during pregnancy was also evaluated. This approach makes it possible to identify a potential cocaine-induced epigenetic effect during early development, prior to exposure to the extrauterine environment.

## Materials and Methods

### Participants

The biological, clinical, and demographic data of the study participants were obtained from a previous cross-sectional study conducted from January 2012 to September 2013 in Southern Brazil, assessing 2,228 births in a public hospital (Mardini et al., 2016). This study evaluated a consecutive sample of crack cocaine-using mothers diagnosed with CUD and their newborns. The research protocols were previously reviewed and authorized by the Ethics Committees of the hospitals. All participants provided written informed consent for participation after receiving information regarding the procedures, objectives, and risks of the study. The use of biological samples and databases was authorized and approved by the Ethics Committee of Pontifical Catholic University of Rio Grande do Sul (PUCRS) under approval number 2.255.141.

### Study Design and Population

In this cross-sectional design study, the factor studied was prenatal crack cocaine exposure, and the outcome was OXTR DNA methylation levels in the UCB of newborns. To find mothers who used crack cocaine during pregnancy, we consecutively interviewed women about their use of illicit and licit substances (i.e., crack cocaine, tobacco, alcohol, and marijuana) upon admission to a public hospital maternity unit in Southern Brazil (*n* = 2,228). Among those reporting crack cocaine use during pregnancy 105 mothers (4.7%) had their use confirmed through positive urine drug tests (Multi-Drogas one-step test, INLAB, Sao Paulo, Brazil) and clinical interviews. All of them were invited to participate in this study and donate UCB from their children. However, only 30 were considered eligible due low DNA quality obtained from UCB, presence of Human Immunodeficiency Virus (HIV) or Hepatitis C Virus infections in mothers, or low quality of clinical data.

The control group (*n* = 30) consisted of newborns not exposed to prenatal crack cocaine, and their UCB samples were obtained from the hospital biobank. Mothers who were invited to donate UCB samples for the hospital biobank had an extensive investigation on exposure to toxic, infectious agents and health records, establishing the absence of any mother/baby pathology. In addition, the control group only included mothers who denied any substance use during pregnancy and showed negative findings in urinary analysis for drugs (Multi-Drogas one-step test, INLAB). The selection of control samples also accounted for matching procedures based on age of mothers who used crack cocaine and were included in the study.

It is important to note that this study focused on the effects of crack (smoked cocaine), instead of snorted/injected cocaine, during gestation. Crack produces all the effects of cocaine. However, its smoked route of administration reaches the lung and brain in a few minutes. Thus, its effects happen much faster than those of snorted cocaine. Crack effect lasts for about 5 min, while the effect of injected/snorted cocaine lasts for about 20–45 min. The short duration of the effect of crack is associated with a pattern of consumption of greater quantities. This behavior causes a much faster pattern of addiction compared to snorted or injected cocaine. Finally, many substances in addition to cocaine might be added to crack rocks, to increase the profit of drug dealers. Crack contains a mixture of cocaine with sodium bicarbonate or ammonia, powdered milk, and lime, in addition to other substances that enhance its neurotoxic effects, such as lidocaine, phenacetin, levamisole, benzocaine, procaine, and hydroxyzine. Based on that, we decided to maintain this distinction.

### Instruments and Variables

The study variables were obtained by *OXTR* DNA methylation analyses, interviews, and reviews of medical records. The independent variable was a history of crack cocaine use during gestation. Maternal information, including age, self-reported ethnicity, prenatal care, education, and the socioeconomic level, was collected at the time of enrollment using a standardized questionnaire. The socioeconomic level was dichotomized into “low socioeconomic status” (classes C, D, and E) and “high socioeconomic status” (classes A and B). This was established based on the Criterion of Economic Classification of Brazil (CCEB), which is a standard classification method of socioeconomic status in Brazil. This indicator has five different classes (A, B, C, D, and E) based on parameters that include the physical structure of the residence, the existence and quantity of certain goods (bathrooms, domestic employees, automobiles, microcomputer, dishwasher, fridge, freezer, washes clothes, DVD, microwave, motorcycle, and clothes dryer), access to public services (water supply, paved street, etc.), the education level of the head of the family (illiterate, elementary school, high school, and graduate level), and family income. Participants classified within the “high socioeconomic status” have an estimated monthly income higher than 10390,00 R$ (1853 US$). Ethnicity was dichotomized as caucasians and others. Educational level was dichotomized as “incomplete or completed primary education, or incomplete secondary education” and “completed secondary education, or incomplete or completed higher education.” The following newborn variables were assessed by reviewing medical records: weight, sex, and Apgar scores at 1 and 5 min of life.

Maternal psychopathology was investigated using the Mini International Neuropsychiatric Interview, Brazilian version 5.0.0/DSM-IV/Current (Amorim, 2000). Also, drug use characteristics and addiction severity were assessed using the Smoking and Substance Involvement Screening Test (ASSIST), a structured interview that allows investigators to evaluate a range of domains often affected by alcohol and drug use up until 3 months prior to delivery. We used a validated Brazilian-Portuguese version (Henrique et al., 2004).

### UCB DNA Extraction

All umbilical cord blood samples were collected in BD Vacutainer^®^ tubes with ethylenediaminetetraaceticacid (EDTA) and centrifuged at 4.0 rotations per minute for 10 min at 4°C. Mononuclear cells were lyzed, and DNA was isolated and purified using the Gentra Puregene^®^ Blood Kit (Qiagen, Hilden, Germany), in accordance with the manufacturer’s standard protocol. Genomic DNA was quantified by ultraviolet absorption using NanoDrop^®^ Lite (Thermo Fisher Scientific, Wilmington, United States). DNA purity was estimated (260/280 nm ratio, 2.0). Samples containing approximately 1,000 ng DNA were stored at −20^°^C for subsequent DNA methylation analysis.

### DNA Methylation Analysis

Sodium bisulfite modification was performed using 1,000 ng of genomic DNA using the EpiTect^®^ Bisulfite Kit (Qiagen) according to the manufacturer’s standard protocol. After bisulfite treatment and purification, the concentration of DNA was reevaluated using NanoDrop^®^ Lite (Thermo Scientific). Bisulfite-converted DNA was used as a template in a polymerase chain reaction (PCR) for the amplification of two target sequences located in exon III of the *OXTR* gene. For this, we used two PyroMark CpG assays (OXTR1, cat. no. PM00016821 and OXTR2, cat. no. PM00016828; Qiagen) according to the manufacturer’s instructions.

**FIGURE 1:**
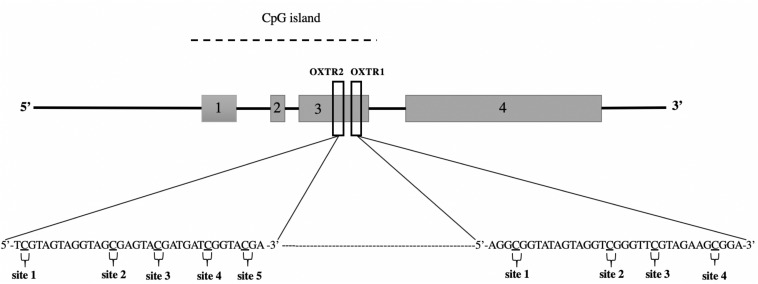
Schematic view of the oxytocin receptor gene (*OXTR*) located at chromosome 3p25, nt 8,809,533–8,809,566. CpG sites within the target sequence analyzed after sequencing are underlined and numbered consecutively. Sites 1–9 indicate the CpG position analyzed by commercial assays, OXTR1 (PM00016821) and OXTR2 (PM00016828).

The PyroMark CpG assay OXTR1 allows analysis of the sequence AGGCGGTATAGTAGGTCGGGTTCGTAG AAGCGGA (Chr:3p25, nt 8809467–8809658; Figure 1). This sequence is a region within exon III of *OXTR* containing four CpG sites (proximal to the 3′ end of the exon). The PCR primer sequences for OXTR1 were as follows: forward, 5′-TTTAGGG ATATGAGTAGTAGTAGGTAGG-3′ and reverse, 5′-ACCCCTCTTCTTCTTCTTCATAAAACACC-3′, which generated a 193-bp amplicon. Similarly, the OXTR2 assay allows analysis of the sequence TCGTAGTAGGTAG CGAGTACGATGATCGGTACGA (Chr:3p25, nt 8,809,533–8,809,566; Figure 1) located within exon III of *OXTR*. This region contains five CpG sites (at the center of the exon). The OXTR2 PCR primers generate a 248-bp amplicon (forward, 5′-GAGGATTTTGGTTTTGGAGATGAGTT-3′; reverse, 5′-CTTC ATCCAACCCTAA AAACCCA-3′). Before the experiment, the PCR primer sets were tested thoroughly to determine reaction efficiency and specificity, and the absence of primer-dimer products.

A 50-ng sample of bisulfite-converted DNA was used as the starting template in a PCR volume of 25 μL using the PyroMark PCR Kit (Qiagen). The PCR program cycle was as follows: 95°C for 15 min; 42 cycles at 95°C for 30 s, 56°C for 30 s, and 72°C for 30 s; and a final extension at 72°C for 5 min. In each plate, we added 0.3 μM of the primer in 25 μL of annealing buffer per well. Further, for each run, we added a blank control (distilled water), a fully methylated positive control (EpiTect^®^ Control Methylated DNA), and a fully unmethylated negative control (EpiTect^®^ Control Unmethylated DNA) to standardize and test the quality of the reactions. Successful PCR amplification of a single fragment was confirmed using agarose gel electrophoresis for each sample. Finally, we prepared single-stranded biotinylated PCR products for sequencing using the Pyrosequencing Vacuum Prep Tool (Qiagen) in accordance with the manufacturer’s instructions.

Polymerase chain reaction products were purified using Streptavidin Sepharose^®^ High Performance (GE Healthcare Europe, Netherlands) followed by hybridization of the pyrosequencing primer in a PyroMark^®^ Q24 Vacuum Workstation (Qiagen). Pyrosequencing was performed using PyroMark^®^ Gold Q24 Reagents (Qiagen) in accordance with the manufacturer’s instructions. We analyzed the data using PyroMark^®^ CpG software version 2.0.6 (Qiagen), which identifies cytosine methylation (%) at each CpG unit within a given sequence. The PyroMark^®^ CpG software generates a quality score for each CpG site throughout the sequencing run, which allowed us to exclude low-quality samples from the pyrosequencing analysis.

In summary, the DNA is first incubated with sodium bisulfite. As a result, unmethylated cytosine residues are converted into uracil, while methylated cytosines remain unchanged, giving rise to two different sequences that can be distinguished. Cytosines that are not methylated are converted to thymine by bisulfite treatment and PCR. Then, in the resulting report, the ratio of cytosine to thymine at each analyzed CpG site is presented, which reflects the proportion of methylated DNA.

Following pyrosequencing analysis, two patients were excluded because of low-quality scores in the methylation analyses. Thus, the final sample consisted of 28 prenatal crack cocaine-exposed newborns and 30 non-exposed newborns.

### Statistical Analysis

All variables were tested for normality of distribution by the Shapiro–Wilk test. Sociodemographic and clinical data were compared between crack cocaine user mothers and non-user mothers, or between crack cocaine-exposed and non-exposed children using the Student’s *t*-test or by using the Chi-squared test for categorical variables. DNA methylation data in the region denominated as OXTR1 were analyzed by calculating the mean values for four CpG units (OXTR1 methylation). A similar calculation was performed for the region denominated as OXTR2, which consisted of the mean values for five CpG units (OXTR2 methylation). Zero-order correlation was applied to explore the relationship between OXTR1 methylation and OXTR2 methylation and clinical and sociodemographic variables (children’s and maternal variables). For DNA methylation prediction analysis, a stepwise linear regression analysis was conducted with OXTR1 methylation and OXTR2 methylation as independent variables and the ASSIST crack cocaine score as the dependent variable. For *p*-value corrections, we used the Bonferroni test. Statistical analysis was performed using SPSS 20.0 (IBM, Chicago, IL, United States). The level of significance was set at *p* ≤ 0.05.

## Results

### Maternal and Infants’ Sociodemographic and Clinical Characteristics

Sociodemographic and clinical characteristics of PCE newborns and non-exposed to cocaine (NEC) newborns, as well as their respective mothers, are shown in Table 1. Both groups were homogeneous concerning children’s birth weight, sex, ethnicity, and 1- and 5-min Apgar scores. Similarly, compared with the non-user mothers (NUMs), the crack cocaine-using mothers (CCUMs) were also mostly non-white. In addition, CCUMs completed less than high school education, were less likely to have received prenatal care, and showed higher scores in psychopathological (except alcohol and drugs) evaluations in comparison with the control group.

**TABLE 1:** Sociodemographic and clinical characteristics of pregnant crack cocaine users and their newborns in comparison to a control group.

**Infant variable**	**NEC(*N* = 30)**	**PCE(*N* = 28)**	**Test*X^2^/t***	***df***	***p***	**Effect size/OR (95% CI)**
Ethnicity						
Caucasians	19 (63.3%)	14 (46.7%)	*1.684^*a*^*	1	0.194	0.50 (0.18–1.42)
Others	11 (36.7%)	16 (53.3%)				
Apgar, 1 min	8.60 ± 1.37	8.48 ± 0.65	*0.406^*b*^*	56	0.684	0.96
Apgar, 5 min	9.63 ± 0.55	9.33 ± 0.63	*1.922^*b*^*	56	0.059	0.94
Weight, kg	3.10 ± 0.4	4.35 ± 0.8	*2.170^*b*^*	57	0.391	1.90
Male sex, *N*	16 (53.3%)	8 (26.7%)	*7.167^*a*^*	*5*	*0.209*	0.43 (0.21–1.16)

**Maternal variable**	**NUM**	**CCUM**	**Test *X^2^/t***	***df***	***p***	**Effect size/OR (95% CI)**

Age, years	26.97 ± 6.18	27.29 (± 6.18)	*0.092^*b*^*	*56*	*0.927*	0.20
Ethnicity						
Caucasians	23 (76.7%)	9 (23.3%)	*8.297^*a*^*	1	***0.004***	4.92 (1.61–15.07)
Others	7 (40.0%)	18 (60.0%)				
Education						
Up to secondary	14 (46.7%)	24 (82.8%)				
Above secondary	16 (53.3%)	5 (17.2%)	*8.379^*a*^*	*1*	***0.004***	5.48 (1.65–18.23)
Prenatal care	30 (100%)	20 (69.0%)	*10.986^*a*^*	*1*	***0.001***	1.45 (1.13–1.85)
Socioeconomic status						
High	8 (26.7%)	2 (6.7%)	*4.320^*a*^*	*1*	***0.038***	5.09 (0.98–26.43)
Low	22 (73.3%)	28 (93.3%)				
Any psychopathology (except alcohol and drugs)	0 (0%)	2 (6.7%)	*2.069^*a*^*	*1*	*0.150*	1.07 (0.97–1.17)

*NUM, non-user mothers; CCUM, crack cocaine-using mothers; PCE, prenatal cocaine-exposed newborns; NEC, non-exposed to cocaine newborns; df, degrees of freedom; g, grams; socioeconomic status high, class a or b; socioeconomic status low, class c, d, or e; education up to secondary, incomplete or completed primary education, or incomplete secondary education; education above secondary, completed secondary education, or incomplete or completed higher education. Data are presented as mean and standard deviation or number of participants and percentage. For t-tests, the effect size reported refers to Cohen’s d. For Chi-squared tests, instead of effect size, the odds ratio is reported with the lower and upper limits and r equivalent to d.*

*^*a*^Chi-squared.*

*^*b*^t-test value.*

*Bold *p* values are significants.*

Mothers of NEC newborns showed an absence of substance abuse (alcohol, nicotine, cannabis, cocaine, or any other substances) throughout their lives. Therefore, we analyzed the ASSIST total scores for alcohol (8.10 ± 7.89), nicotine (15.17 ± 8.97), cannabis (6.4 ± 8.71), and crack cocaine (25.53 ± 8.52) usage within CCUMs. Further, we examined the frequency of drug use during the 3 months prior to delivery (Table 2).

**TABLE 2:** Frequency of drug use by crack cocaine-using mothers.

**Substance**	**Daily**	**Weekly**	**Monthly**	**Once or twice**	**Never**
Nicotine (%)	14 (48.2%)	2 (6.7%)	3 (10.0%)	2 (7.4%)	8 (27.7%)
Alcohol (%)	3 (10.3%)	6 (20.7%)	4 (12.6%)	1 (5.0%)	15 (48.4%)
Cannabis (%)	4 (13.3%)	3 (10%)	2 (6.7%)	2 (6.7%)	17 (60%)
Crack cocaine (%)	11 (36.7%)	15 (53.3%)	3 (10%)	1 (5.0%)	0 (0%)

*Data are presented as number of participants and percentage.*

### DNA Methylation Levels in UCB

The OXTR1, OXTR2, and overall OXTR DNA methylation data of PCE and NEC newborns are shown in Table 3. No differences in DNA methylation levels were observed between case and control children.

**TABLE 3:** DNA methylation levels in *OXTR* exon III in the umbilical cord blood of the prenatal cocaine-exposed and non-exposed newborns.

**OXTR assay**	**NEC (*n* = 30) Mean ± SD**	**PCE (*n* = 28) Mean ± SD**	**Test**	***df***	**Corrected *p***	**Effect size**
OXTR1 met	22.04 ± 21.54	19.08 ± 17.44	*t* = *0.571*	*56*	*0.570*	0.16
OXTR2 met	26.87 ± 21.87	22.05 ± 13.33	*t* = *0.950*	50	*0.347*	0.30
OXTR overall	24.14 ± 15.45	19.98 ± 13.23	*t* = *1.097*	56	*0.277*	0.27

*PCE, prenatal cocaine-exposed newborns; NEC, non-exposed to cocaine newborns; OXTR1 met, OXTR1 methylation; OXTR2 met, OXTR2 methylation; CpG, cytosine-phosphate-guanine. Data are presented as mean and standard deviation. Effect size refers to Cohen’s d.*

### Relationships Between OXTR DNA Methylation and Sociodemographic Characteristics and ASSIST Scores

We performed exploratory zero-order correlation analyses to investigate the relationship between *OXTR* methylation and maternal and neonatal sociodemographic and clinical variables. OXTR1 and OXTR2 methylation levels of the PCE newborns were not associated with Apgar scores at 1 and 5 min; newborn weight; maternal age; ASSIST scores for tobacco, alcohol, or cannabis (Table 4); or other sociodemographic and clinical variables (all *p* > 0.05). Additionally, OXTR methylation was compared between groups of socioeconomic status (High vs. Low; OXTR1–*t* = 0.75; *p* = 0.45; OXTR2–*t* = 0.72; *p* = 0.47), ethnicity (Caucasian vs. Others; OXTR1–*t* = 0.02; *p* = 0.98; OXTR2–*t* = 0.17; *p* = 0.86), and education (up to secondary vs. Above secondary; OXTR1–*t* = 0.10; *p* = 0.91; OXTR2–*t* = 1.08; *p* = 0.28), showing that methylation levels also were not associated with these variables. However, OXTR1 methylation showed a moderate correlation with crack cocaine ASSIST scores (Table 4).

**TABLE 4:** Zero-order correlations.

**Variable**	**Apgar 1 (min)**	**Apgar 5 (min)**	**Children weight (g)**	**Maternal age (years)**	**ASSIST tobacco**	**ASSIST alcohol**	**ASSIST cannabis**	**ASSIST crack cocaine**	**OXTR1 methylation**	**OXTR2 methylation**
Apgar 1 (min)	–									
Apgar 5 (min)	0.66**	–								
Children weight (g)	−0.23	−43*	–							
Maternal age (years)	0.31	0.09	−0.24	–						
ASSIST tobacco	0.13	0.04	0.11	0.16	–					
ASSIST alcohol	−0.01	0.10	−0.13	−0.08	0.06	–				
ASSIST cannabis	0.20	0.21	−0.13	−0.16	0.02	−0.02	–			
ASSIST crack cocaine	−0.15	−0.05	0.07	−0.51	−0.13	0.11	−0.06	–		
OXTR1 methylation	−0.14	−0.28	0.09	0.05	−0.08	−0.01	−0.13	0.36*	–	
OXTR2 methylation	0.19	0.22	0.04	−0.11	0.24	0.20	0.19	0.01	−0.12	–

*Apgar 1, Apgar score at 1 min; Apgar 5, Apgar score at 5 min; ASSIST, tobacco; ASSIST, scale score for tobacco consumption; ASSIST, cannabis; ASSIST, scale score for cannabis consumption; ASSIST, alcohol; ASSIST, scale score for alcohol consumption; ASSIST, crack cocaine; ASSIST, scale score for crack cocaine consumption.*

i*p ≤ 0.05.

**p ≤ 0.005.

**FIGURE 2:**
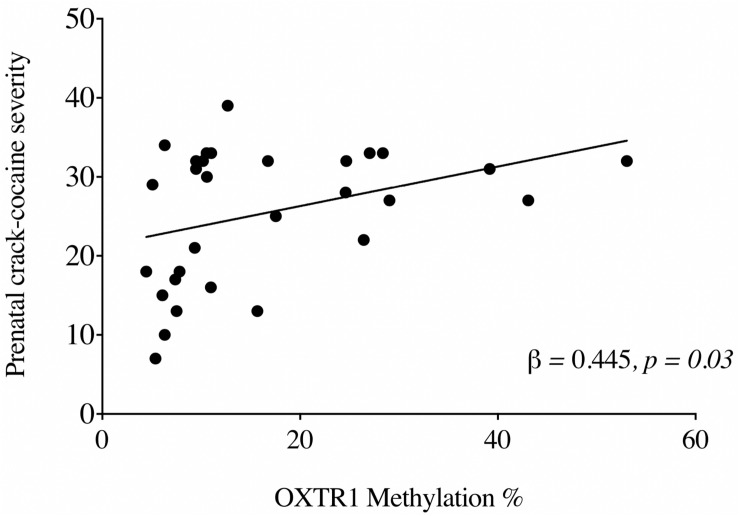
Relationship between levels (%) of *OXTR1* methylation in PCE newborns and ASSIST scores for crack cocaine usage among mothers in the 3 months prior to delivery.

To examine the influence of crack cocaine exposure on UCB *OXTR* methylation, we performed a stepwise linear regression model including OXTR1 methylation and OXTR2 methylation as independent variables and ASSIST score for crack cocaine as a dependent variable. Further, we included the mothers’ age and ethnicity, children’s ethnicity, and ASSIST scores for tobacco, alcohol, and cannabis as covariates. The results showed that maternal ASSIST scores for crack cocaine predicted *OXTR* methylation [*R* = 0.445 *R*^2^ = 0.198; *F*_(__5_,_176__)_ = 0.160, *p* = 0.003] (Figure 2). Only OXTR1 methylation was included in the final regression model (β = 0.445, *p* = 0.03).

## Discussion

This is the first study to investigate DNA methylation of *OXTR* in the UCB of PCE children. We found that maternal ASSIST crack cocaine scores predicted DNA methylation levels of the CpG island within exon III of the *OXTR* gene among PCE children, even after controlling for potential confounders. Although our analysis revealed this positive association, we found no differences between groups (newborns not exposed to crack). These data reveal that the magnitude of the effects of prenatal crack cocaine exposure at the epigenetic level might be dependent on the severity of maternal consumption patterns (Ross et al., 2015).

Studies have reported that increased *OXTR* DNA methylation in the CpG island within exon I–III is associated with decreased gene expression (Kusui et al., 2001) and lower plasma oxytocin levels (Ebner et al., 2019), resulting in decreased oxytocinergic signaling (Kusui et al., 2001). These alterations in *OXTR* expression could be a possible etiological factor for the social and affiliative dysfunction observed in PCE children (Gouin et al., 2017), probably due to the relevance of the OT system in social behavior and early neuronal development (Williams and Johns, 2014). For instance, maternal substance abuse of tobacco, cannabis, and alcohol has been associated with cord blood DNA methylation changes in newborns (Knopik et al., 2019). Furthermore, preclinical investigations documented that prenatal exposure to cocaine leads to brain DNA methylation modifications across the genome, which can be considered an early life insult (Vaiserman, 2013; Kundakovic and Jaric, 2017; Knopik et al., 2019).

The epigenome is dynamic and experiences after birth continue to exert regulation on gene expression through DNA methylation and chromatin modifications (Kraaijenvanger et al., 2019). Therefore, we can hypothesize that prenatal exposure to cocaine may induce subtle epigenetic changes at birth, but with greater influence on DNA methylation levels throughout development. Particularly, it seems that during pregnancy only high levels of crack cocaine consumption can produce detectable changes in *OXTR* methylation, as observed in our linear regression analysis. The mechanism underlying this association might be attributable to the effects that cocaine has on methyl-binding proteins and DNA methyltransferases, which have been substantially documented in preclinical studies (Nestler, 2014). In this sense, many animal studies have described the association between cocaine exposure and DNA methylation changes in the CNS (Im et al., 2010). Cocaine injection in the nucleus accumbens and hippocampus of rats was followed by increased transcription of DNA methyltransferase 3 (DNMT3), which is an enzyme involved in establishing *de novo* cytosine methylation and maintenance in both embryonic and somatic cells (Anier et al., 2013). Likewise, Tian et al. (2012) reported increased mRNA levels of *DNMT3* in the prefrontal cortex of mice after cocaine self-administration. This suggests that cocaine can permanently alter the functioning of genes involved in the molecular machinery of *de novo* DNA methylation establishment, and this might affect *OXTR* gene methylation. This finding has potential implications for vulnerability to mental disorders in humans, given that increased *OXTR* DNA methylation in exon III has been linked to autism spectrum disorder (Gregory et al., 2009), psychopathy (Dadds et al., 2014), obsessive-compulsive disorder (Cappi et al., 2016), depression (Chagnon et al., 2015), and anxiety disorder (Ziegler et al., 2015). This is relevant considering evidence suggesting a correlation between *OXTR* methylation in peripheral tissues and the brain (Dadds et al., 2014; Joubert et al., 2016; Maud et al., 2018). For instance, in a previous study, autistic patients showed similarities in epigenetic profiles related to *OXTR* methylation in two different types of tissues: peripheral blood mononuclear cells and the temporal cortex (Puglia et al., 2018). The extent to which blood-based DNA methylation levels reflect DNA methylation patterns in the brain is unclear. However, we could hypothesize that the levels of *OXTR* methylation in UCB might be consistent with *OXTR* methylation in the brain, especially considering the proximity of the neurodevelopmental period.

Also, PCE may alter the expression of the *OXTR* gene throughout partially distinct epigenetic regulatory mechanisms, given that the *OXTR* has a series of mRNA isoforms and complex transcriptional regulation. For example, changes in the mechanism of regulation of gene expression mediated by the TET1 protein (Tet Methylcytosine Dioxigenase 1) are known to be related to *OXTR* CpG hypermethylation (Towers et al., 2018). Furthermore, previous studies suggest an interaction between DNA modifications and microRNA dysregulation that targets *OXTR* in the brain (Mor et al., 2015). Therefore, in addition to DNA methylation, histone modifications and non-coding RNAs could also be involved in the potential epigenetic effects of PCE on the *OXTR* gene (Feng and Nestler, 2013; Vaiserman, 2013). Due to the complexity of the *OXTR* gene, it is possible that group differences that were not evident in our analyses could be observed in future studies through a broader appreciation of the epigenome.

The ASSIST scale allowed us to measure the severity of psychoactive substance use of the mothers, making it possible to verify the degree of drug addiction for distinct substances (Henrique et al., 2004). In our analyses, most mothers had a history of chronic crack use, with more than half of the sample reporting weekly use (53%) and 36% reporting daily use. Particularly, the rate of crack cocaine use during pregnancy of 4.7% found in our study is in agreement with estimates from other epidemiological studies in Brazil. For instance, a study that analyzed the prevalence of prenatal exposure to cocaine through meconium analysis of 739 newborns found that 4.6% of them were exposed to cocaine (Cunha et al., 2001). Laranjeira et al. (2006) investigated the use of psychoactive substances in 1,000 pregnant adolescents (11–19 years old) in a public hospital in Sao Paulo, finding a prevalence of cocaine and marijuana use of 6% in the third trimester of pregnancy (Mitsuhiro et al., 2006), which is similar to the data reported in our study. In this sense, previous studies have reported similar concentrations of metabolites of numerous substances consumed by mothers (cocaine, opiates, methadone, and amphetamines) in UCB and newborn peripheral blood (Concheiro et al., 2013; Colby, 2017). Moreover, UCB-based assessments are considered a superior method of evaluation of biological alterations during the fetal period when compared to assessments of other types of tissues. UCB is a tissue that provides information about an organism that has not yet experienced direct postnatal exposure to environmental factors that can potentially affect DNA methylation (Lozano et al., 2007). In this regard, several intrauterine predictors of cord blood DNA methylation in human newborns have been identified, including maternal substance use (Joubert et al., 2012, 2016). This is consistent with our data, in which the severity of crack cocaine use was a predictor of *OXTR* methylation in UCB of PCE children.

We have shown ethnic differences between mothers using and not using crack cocaine and between exposed and non-exposed newborn children; however, we found no association between ethnicity and *OXTR* methylation levels, contrary to what has been observed in other studies. For instance, Adkins et al. (2011) examined genomic UCB DNA from healthy newborn African and Caucasian children and reported significantly lower methylation levels at 68% of the CpG sites examined in African newborns. This was similar to the findings obtained by Terry et al. (2008), who reported that adult Africans/Blacks were found to display lower levels of methylation than adult Caucasians/Whites and Hispanics. Interestingly, in both studies, no differences in methylation were observed, even after adjusting for confounders such as smoking, alcohol consumption, and other substance use/exposure. This led us to hypothesize that although ethnicity is a factor known to influence DNA methylation patterns, these effects may be small regarding *OXTR* exon III CpG island.

It must be discussed, however, that there are significant limitations in this study. Although crack cocaine was required to be the drug of choice as an inclusion criterion in the study, the presence of other psychoactive substances was not an exclusion criterion. Previous research indicates that mothers who use cocaine during pregnancy are likely to also use other substances (Lozano et al., 2007; Vaiserman, 2013; Wanner et al., 2019). Without appropriate controls for polydrug exposure, it is difficult to establish more robust conclusions regarding the specific effects of PCE on OXTR methylation because PCE often co-occurs with intrauterine exposure to tobacco, alcohol, and marijuana (Wanner et al., 2019). Another limitation was the small sample size, which could have affected between-group analyses. Furthermore, significant differences in maternal socioeconomic variables between groups were found, which might account for variability in OXTR methylation rather than substance use patterns. However, we performed correlation and group comparisons accounting for maternal ethnicity, education, APGAR, age, weight, and socioeconomic status, and no significant associations were observed regarding DNA methylation levels. We used self-report measures and urine tests for the assessment of crack cocaine use. Particularly, urine tests only capture short-term exposure compared to other methods, such as hair follicle drug test that captures exposure to psychoactive substances up to 6 months. We would like to highlight that investigations of other regions of interest will be desirable in future studies. We did not analyze additional OXTR regions besides exon III. Therefore, additional research should be done to determine whether PCE is able to modulate DNA methylation on distinct exons of the OXTR gene in UCB samples, including promoter regions, SNP regions, and regions regulated by non-coding RNAs. Further, the heterogeneous mixture of cell types in the UCB samples used for methylation analyses may have constituted a potential confounder. In addition, we could not explore correlation with OXTR RNA- or protein changes, and we cannot conclude whether the observed DNA-methylation finding is functionally relevant. Therefore, the present findings should be interpreted as well-grounded hypotheses for further examination in larger studies.

## Conclusion

We provide preliminary evidence that the mother’s severity of crack use is associated with an increase in OXTR DNA methylation. These results provide additional evidence to the potential role of DNA methylation in the relationship between maternal substance use and later behavioral and developmental sequelae in offspring (Knopik et al., 2019). Particularly, DNA methylation could be one of the mechanisms by which cocaine disrupts the oxytocinergic system. Also, considering the prosocial and affective role of oxytocin, epigenetic changes that might result in altered expression of the OXTR gene may influence the development of the child.

## Data Availability Statement

The raw data supporting the conclusions of this article will be made available by the authors, without undue reservation.

## Ethics Statement

The studies involving human participants were reviewed and approved by Pontifícia Universidade Católica do Rio Grande do Sul. Written informed consent to participate in this study was provided by the participants’ legal guardian/next of kin.

## Author Contributions

TB and RG-O conceived and planned the experiments. TB and LA carried out the experiments. MN, AP, and FM carried out pyrosequencing procedures. VM and CS contributed to sample recruitment and clinical assessment. TV, SC, and JS contributed to the interpretation of the results. TB took the lead in writing the manuscript. RG-O was in charge of the overall direction and plan, and supervised the findings of this work. All authors provided critical feedback and helped shape the research, analysis, and manuscript.

## Conflict of Interest

The authors declare that the research was conducted in the absence of any commercial or financial relationships that could be construed as a potential conflict of interest.
